# Supplementary material for: Multi‐dataset identification of innovative feature genes and molecular mechanisms in keratoconus
Source: J Cell Mol Med. 2024 Sep 19;28(18):e70079. doi: 10.1111/jcmm.70079 (PMC11412914; doi:10.1111/jcmm.70079)
Supplement: Supplementary file 2 — Material S2. [file JCMM-28-e70079-s002.zip › 7.GSEA analysis/4.MYRF/KEGG_PATHWAYS_IN_CANCER.html]

Details for gene set KEGG\_PATHWAYS\_IN\_CANCER[GSEA]

|  || Dataset | MYRF.group.cls#h\_versus\_l |
| Phenotype | group.cls#h\_versus\_l |
| Upregulated in class | l |
| GeneSet | KEGG\_PATHWAYS\_IN\_CANCER |
| Enrichment Score (ES) | -0.401474 |
| Normalized Enrichment Score (NES) | -1.7394955 |
| Nominal p-value | 0.0020120724 |
| FDR q-value | 0.21462911 |
| FWER p-Value | 0.301 |
Table: GSEA Results Summary

  

Fig 1: Enrichment plot: KEGG\_PATHWAYS\_IN\_CANCER      
 Profile of the Running ES Score & Positions of GeneSet Members on the Rank Ordered List

  

| SYMBOL | TITLE | RANK IN GENE LIST | RANK METRIC SCORE | RUNNING ES | CORE ENRICHMENT || 1 | PIK3R5 | na | 661 | 0.528 | -0.0079 | No |
| 2 | PDGFRA | na | 1428 | 0.407 | -0.0214 | No |
| 3 | AR | na | 1716 | 0.381 | -0.0216 | No |
| 4 | RAC3 | na | 2468 | 0.325 | -0.0365 | No |
| 5 | CCNE2 | na | 2609 | 0.317 | -0.0338 | No |
| 6 | FGF8 | na | 2858 | 0.303 | -0.0345 | No |
| 7 | RARB | na | 3128 | 0.289 | -0.0361 | No |
| 8 | RET | na | 3508 | 0.271 | -0.0413 | No |
| 9 | CBLB | na | 3959 | 0.256 | -0.0489 | No |
| 10 | LAMB4 | na | 3997 | 0.254 | -0.0446 | No |
| 11 | FLT3 | na | 5327 | 0.212 | -0.0786 | No |
| 12 | NTRK1 | na | 5375 | 0.211 | -0.0755 | No |
| 13 | FGF18 | na | 5499 | 0.207 | -0.0746 | No |
| 14 | WNT10B | na | 5576 | 0.206 | -0.0724 | No |
| 15 | FGF16 | na | 5776 | 0.199 | -0.0739 | No |
| 16 | SPI1 | na | 5859 | 0.197 | -0.0721 | No |
| 17 | PTCH1 | na | 6108 | 0.189 | -0.0753 | No |
| 18 | KLK3 | na | 6644 | 0.174 | -0.0871 | No |
| 19 | SOS1 | na | 6706 | 0.172 | -0.0852 | No |
| 20 | FN1 | na | 6837 | 0.168 | -0.0853 | No |
| 21 | DAPK1 | na | 7051 | 0.162 | -0.0881 | No |
| 22 | PIK3R1 | na | 7105 | 0.161 | -0.0861 | No |
| 23 | IGF1R | na | 7135 | 0.160 | -0.0836 | No |
| 24 | MECOM | na | 7274 | 0.157 | -0.0842 | No |
| 25 | FGF5 | na | 7739 | 0.145 | -0.0946 | No |
| 26 | APC2 | na | 8076 | 0.137 | -0.1014 | No |
| 27 | FGF3 | na | 8177 | 0.134 | -0.1015 | No |
| 28 | BRAF | na | 8198 | 0.133 | -0.0992 | No |
| 29 | COL4A1 | na | 8213 | 0.133 | -0.0968 | No |
| 30 | MAPK9 | na | 8409 | 0.129 | -0.0997 | No |
| 31 | MTOR | na | 8431 | 0.129 | -0.0975 | No |
| 32 | WNT16 | na | 8449 | 0.128 | -0.0953 | No |
| 33 | IGF1 | na | 8743 | 0.121 | -0.1012 | No |
| 34 | FGF12 | na | 8823 | 0.120 | -0.1009 | No |
| 35 | CASP3 | na | 8824 | 0.120 | -0.0983 | No |
| 36 | FZD1 | na | 9008 | 0.116 | -0.1012 | No |
| 37 | WNT5A | na | 9048 | 0.115 | -0.0998 | No |
| 38 | CCNA1 | na | 9171 | 0.112 | -0.1010 | No |
| 39 | CASP8 | na | 9334 | 0.109 | -0.1034 | No |
| 40 | KRAS | na | 9371 | 0.108 | -0.1021 | No |
| 41 | CREBBP | na | 9688 | 0.102 | -0.1091 | No |
| 42 | PIK3CG | na | 9794 | 0.100 | -0.1100 | No |
| 43 | CBL | na | 9933 | 0.097 | -0.1119 | No |
| 44 | TRAF3 | na | 10032 | 0.095 | -0.1128 | No |
| 45 | FZD3 | na | 10112 | 0.094 | -0.1130 | No |
| 46 | AKT2 | na | 10210 | 0.092 | -0.1139 | No |
| 47 | FGF17 | na | 10236 | 0.092 | -0.1126 | No |
| 48 | FGF13 | na | 10299 | 0.091 | -0.1125 | No |
| 49 | CEBPA | na | 10512 | 0.086 | -0.1168 | No |
| 50 | FADD | na | 10767 | 0.082 | -0.1224 | No |
| 51 | MSH3 | na | 11107 | 0.076 | -0.1307 | No |
| 52 | PTEN | na | 11228 | 0.075 | -0.1326 | No |
| 53 | CCNE1 | na | 11231 | 0.075 | -0.1310 | No |
| 54 | PIK3CA | na | 11255 | 0.074 | -0.1301 | No |
| 55 | E2F3 | na | 11256 | 0.074 | -0.1285 | No |
| 56 | LAMA5 | na | 11263 | 0.074 | -0.1271 | No |
| 57 | VHL | na | 11380 | 0.072 | -0.1289 | No |
| 58 | ARNT | na | 11576 | 0.069 | -0.1331 | No |
| 59 | XIAP | na | 11781 | 0.066 | -0.1376 | No |
| 60 | SUFU | na | 11793 | 0.066 | -0.1365 | No |
| 61 | FZD5 | na | 11924 | 0.065 | -0.1389 | No |
| 62 | PIAS1 | na | 11967 | 0.064 | -0.1388 | No |
| 63 | LAMC3 | na | 12089 | 0.062 | -0.1409 | No |
| 64 | RXRA | na | 12183 | 0.061 | -0.1423 | No |
| 65 | HSP90AA1 | na | 12360 | 0.059 | -0.1462 | No |
| 66 | MAPK10 | na | 12440 | 0.057 | -0.1473 | No |
| 67 | WNT3 | na | 12453 | 0.057 | -0.1464 | No |
| 68 | E2F2 | na | 12461 | 0.057 | -0.1454 | No |
| 69 | PIK3R2 | na | 12535 | 0.056 | -0.1463 | No |
| 70 | STAT1 | na | 12606 | 0.055 | -0.1472 | No |
| 71 | RB1 | na | 12759 | 0.053 | -0.1504 | No |
| 72 | SOS2 | na | 12783 | 0.053 | -0.1500 | No |
| 73 | APPL1 | na | 12790 | 0.053 | -0.1490 | No |
| 74 | FGF20 | na | 12795 | 0.053 | -0.1480 | No |
| 75 | PLCG1 | na | 12816 | 0.052 | -0.1475 | No |
| 76 | TP53 | na | 12977 | 0.051 | -0.1510 | No |
| 77 | PLCG2 | na | 12987 | 0.050 | -0.1502 | No |
| 78 | IKBKB | na | 12994 | 0.050 | -0.1493 | No |
| 79 | ERBB2 | na | 13228 | 0.047 | -0.1551 | No |
| 80 | BID | na | 13268 | 0.047 | -0.1552 | No |
| 81 | TRAF2 | na | 13353 | 0.046 | -0.1567 | No |
| 82 | SMAD4 | na | 13356 | 0.045 | -0.1558 | No |
| 83 | FZD4 | na | 13390 | 0.045 | -0.1558 | No |
| 84 | WNT2 | na | 13551 | 0.043 | -0.1595 | No |
| 85 | RALBP1 | na | 13577 | 0.043 | -0.1593 | No |
| 86 | MDM2 | na | 13578 | 0.043 | -0.1584 | No |
| 87 | FGF9 | na | 13888 | 0.039 | -0.1665 | No |
| 88 | PDGFA | na | 13993 | 0.038 | -0.1687 | No |
| 89 | MAP2K2 | na | 14012 | 0.038 | -0.1684 | No |
| 90 | CDKN1B | na | 14078 | 0.037 | -0.1695 | No |
| 91 | RALGDS | na | 14160 | 0.036 | -0.1711 | No |
| 92 | PRKCA | na | 14202 | 0.036 | -0.1715 | No |
| 93 | NCOA4 | na | 14233 | 0.035 | -0.1717 | No |
| 94 | FH | na | 14305 | 0.034 | -0.1730 | No |
| 95 | CHUK | na | 14348 | 0.034 | -0.1735 | No |
| 96 | MAPK3 | na | 14382 | 0.034 | -0.1737 | No |
| 97 | CBLC | na | 14386 | 0.034 | -0.1731 | No |
| 98 | FGFR3 | na | 14422 | 0.033 | -0.1734 | No |
| 99 | CTBP2 | na | 14461 | 0.033 | -0.1738 | No |
| 100 | CTNNA2 | na | 14571 | 0.032 | -0.1763 | No |
| 101 | CCND1 | na | 14657 | 0.031 | -0.1781 | No |
| 102 | RBX1 | na | 14789 | 0.030 | -0.1813 | No |
| 103 | CTBP1 | na | 14847 | 0.029 | -0.1823 | No |
| 104 | TCF7L2 | na | 15138 | 0.027 | -0.1902 | No |
| 105 | PTCH2 | na | 15175 | 0.026 | -0.1906 | No |
| 106 | STAT5B | na | 15287 | 0.025 | -0.1933 | No |
| 107 | FZD9 | na | 15491 | 0.023 | -0.1987 | No |
| 108 | BCR | na | 15503 | 0.023 | -0.1986 | No |
| 109 | FGF7 | na | 15518 | 0.023 | -0.1985 | No |
| 110 | CTNNB1 | na | 15600 | 0.022 | -0.2004 | No |
| 111 | HHIP | na | 15667 | 0.021 | -0.2018 | No |
| 112 | CDH1 | na | 15708 | 0.021 | -0.2026 | No |
| 113 | PRKCG | na | 15973 | 0.018 | -0.2098 | No |
| 114 | PIAS4 | na | 16014 | 0.018 | -0.2106 | No |
| 115 | FASLG | na | 16299 | 0.015 | -0.2186 | No |
| 116 | CTNNA1 | na | 16494 | 0.013 | -0.2239 | No |
| 117 | DAPK3 | na | 16530 | 0.013 | -0.2247 | No |
| 118 | JUN | na | 16671 | 0.011 | -0.2285 | No |
| 119 | EGFR | na | 17031 | 0.008 | -0.2388 | No |
| 120 | PIK3CB | na | 17199 | 0.007 | -0.2435 | No |
| 121 | RAC2 | na | 17259 | 0.006 | -0.2450 | No |
| 122 | MAX | na | 17507 | 0.004 | -0.2521 | No |
| 123 | RALA | na | 17556 | 0.004 | -0.2535 | No |
| 124 | SMAD3 | na | 17747 | 0.002 | -0.2589 | No |
| 125 | HDAC2 | na | 17814 | 0.001 | -0.2608 | No |
| 126 | PLD1 | na | 17905 | 0.000 | -0.2634 | No |
| 127 | WNT8A | na | 17911 | 0.000 | -0.2636 | No |
| 128 | BAX | na | 17915 | 0.000 | -0.2637 | No |
| 129 | MSH2 | na | 18082 | -0.001 | -0.2685 | No |
| 130 | SMAD2 | na | 18091 | -0.001 | -0.2687 | No |
| 131 | AKT1 | na | 18101 | -0.001 | -0.2689 | No |
| 132 | CCDC6 | na | 18111 | -0.002 | -0.2691 | No |
| 133 | GSTP1 | na | 18399 | -0.004 | -0.2774 | No |
| 134 | CSF3R | na | 18485 | -0.005 | -0.2797 | No |
| 135 | STK4 | na | 18494 | -0.005 | -0.2799 | No |
| 136 | PPARD | na | 18532 | -0.006 | -0.2808 | No |
| 137 | BIRC2 | na | 18537 | -0.006 | -0.2808 | No |
| 138 | FGF19 | na | 18545 | -0.006 | -0.2809 | No |
| 139 | RALB | na | 18549 | -0.006 | -0.2809 | No |
| 140 | MAPK8 | na | 18612 | -0.006 | -0.2825 | No |
| 141 | RASSF5 | na | 18628 | -0.007 | -0.2828 | No |
| 142 | FGF10 | na | 18767 | -0.008 | -0.2867 | No |
| 143 | RHOA | na | 18827 | -0.008 | -0.2882 | No |
| 144 | EGF | na | 19142 | -0.012 | -0.2971 | No |
| 145 | PIAS3 | na | 19292 | -0.014 | -0.3011 | No |
| 146 | GSK3B | na | 19466 | -0.016 | -0.3058 | No |
| 147 | HDAC1 | na | 19552 | -0.017 | -0.3079 | No |
| 148 | FZD6 | na | 19569 | -0.017 | -0.3080 | No |
| 149 | FGF11 | na | 19615 | -0.017 | -0.3089 | No |
| 150 | CDKN2A | na | 19779 | -0.019 | -0.3133 | No |
| 151 | JUP | na | 19857 | -0.020 | -0.3151 | No |
| 152 | RASSF1 | na | 20045 | -0.022 | -0.3200 | No |
| 153 | FZD8 | na | 20046 | -0.022 | -0.3196 | No |
| 154 | JAK1 | na | 20051 | -0.022 | -0.3192 | No |
| 155 | TRAF4 | na | 20246 | -0.025 | -0.3243 | No |
| 156 | VEGFB | na | 20319 | -0.026 | -0.3258 | No |
| 157 | MAPK1 | na | 20406 | -0.027 | -0.3278 | No |
| 158 | PIK3CD | na | 20433 | -0.027 | -0.3280 | No |
| 159 | TPM3 | na | 20434 | -0.027 | -0.3274 | No |
| 160 | PIK3R3 | na | 20464 | -0.027 | -0.3276 | No |
| 161 | FOS | na | 20589 | -0.029 | -0.3306 | No |
| 162 | FGF14 | na | 20664 | -0.030 | -0.3321 | No |
| 163 | HSP90AB1 | na | 20726 | -0.030 | -0.3333 | No |
| 164 | WNT4 | na | 20821 | -0.031 | -0.3353 | No |
| 165 | WNT3A | na | 20924 | -0.033 | -0.3376 | No |
| 166 | FGFR2 | na | 20983 | -0.033 | -0.3386 | No |
| 167 | MSH6 | na | 21088 | -0.035 | -0.3409 | No |
| 168 | TGFA | na | 21199 | -0.036 | -0.3433 | No |
| 169 | WNT9A | na | 21202 | -0.036 | -0.3426 | No |
| 170 | PAX8 | na | 21623 | -0.041 | -0.3539 | No |
| 171 | KIT | na | 21671 | -0.042 | -0.3544 | No |
| 172 | IKBKG | na | 21687 | -0.042 | -0.3539 | No |
| 173 | RAF1 | na | 21720 | -0.042 | -0.3539 | No |
| 174 | E2F1 | na | 21781 | -0.043 | -0.3548 | No |
| 175 | CDKN2B | na | 21823 | -0.044 | -0.3550 | No |
| 176 | GLI3 | na | 21836 | -0.044 | -0.3544 | No |
| 177 | HRAS | na | 21856 | -0.044 | -0.3540 | No |
| 178 | FLT3LG | na | 21921 | -0.045 | -0.3550 | No |
| 179 | RAC1 | na | 21936 | -0.045 | -0.3544 | No |
| 180 | LAMB2 | na | 21964 | -0.045 | -0.3542 | No |
| 181 | TGFB2 | na | 22177 | -0.048 | -0.3593 | No |
| 182 | BIRC5 | na | 22281 | -0.049 | -0.3613 | No |
| 183 | DVL1 | na | 22373 | -0.050 | -0.3628 | No |
| 184 | SKP2 | na | 22430 | -0.051 | -0.3634 | No |
| 185 | DAPK2 | na | 22587 | -0.053 | -0.3668 | No |
| 186 | CASP9 | na | 23052 | -0.060 | -0.3790 | No |
| 187 | FOXO1 | na | 23099 | -0.061 | -0.3790 | No |
| 188 | LAMA2 | na | 23159 | -0.062 | -0.3794 | No |
| 189 | DVL3 | na | 23247 | -0.063 | -0.3805 | No |
| 190 | CDC42 | na | 23297 | -0.064 | -0.3806 | No |
| 191 | AXIN1 | na | 23328 | -0.065 | -0.3801 | No |
| 192 | EGLN1 | na | 23393 | -0.066 | -0.3805 | No |
| 193 | MLH1 | na | 23522 | -0.068 | -0.3828 | No |
| 194 | TRAF6 | na | 23526 | -0.068 | -0.3814 | No |
| 195 | CYCS | na | 23572 | -0.069 | -0.3813 | No |
| 196 | KITLG | na | 23576 | -0.069 | -0.3799 | No |
| 197 | TPR | na | 23610 | -0.069 | -0.3794 | No |
| 198 | PTK2 | na | 23650 | -0.070 | -0.3790 | No |
| 199 | DCC | na | 23704 | -0.071 | -0.3790 | No |
| 200 | ITGA3 | na | 23712 | -0.071 | -0.3777 | No |
| 201 | BAD | na | 23795 | -0.072 | -0.3785 | No |
| 202 | EGLN2 | na | 23813 | -0.073 | -0.3775 | No |
| 203 | ABL1 | na | 23912 | -0.074 | -0.3787 | No |
| 204 | RARA | na | 23932 | -0.074 | -0.3777 | No |
| 205 | LAMB1 | na | 24119 | -0.078 | -0.3814 | No |
| 206 | GRB2 | na | 24215 | -0.079 | -0.3825 | No |
| 207 | CKS1B | na | 24260 | -0.080 | -0.3821 | No |
| 208 | BIRC3 | na | 24271 | -0.080 | -0.3806 | No |
| 209 | EPAS1 | na | 24322 | -0.081 | -0.3804 | No |
| 210 | FGF22 | na | 24497 | -0.084 | -0.3836 | No |
| 211 | CDK4 | na | 24674 | -0.088 | -0.3869 | No |
| 212 | HSP90B1 | na | 24682 | -0.088 | -0.3852 | No |
| 213 | HIF1A | na | 24865 | -0.091 | -0.3885 | No |
| 214 | TGFBR2 | na | 24993 | -0.093 | -0.3902 | No |
| 215 | TFG | na | 25000 | -0.093 | -0.3884 | No |
| 216 | HGF | na | 25029 | -0.093 | -0.3872 | No |
| 217 | SMO | na | 25095 | -0.095 | -0.3871 | No |
| 218 | PML | na | 25100 | -0.095 | -0.3852 | No |
| 219 | ITGA2 | na | 25150 | -0.096 | -0.3845 | No |
| 220 | CTNNA3 | na | 25302 | -0.099 | -0.3868 | No |
| 221 | CRK | na | 25398 | -0.101 | -0.3874 | No |
| 222 | BRCA2 | na | 25524 | -0.103 | -0.3888 | No |
| 223 | TGFBR1 | na | 25528 | -0.103 | -0.3867 | No |
| 224 | CUL2 | na | 25599 | -0.105 | -0.3865 | No |
| 225 | PRKCB | na | 25770 | -0.108 | -0.3891 | No |
| 226 | CDK6 | na | 25789 | -0.108 | -0.3873 | No |
| 227 | RXRB | na | 25902 | -0.111 | -0.3882 | No |
| 228 | ITGA2B | na | 25913 | -0.111 | -0.3861 | No |
| 229 | STAT5A | na | 25954 | -0.112 | -0.3849 | No |
| 230 | COL4A2 | na | 26047 | -0.114 | -0.3851 | No |
| 231 | LAMA4 | na | 26157 | -0.116 | -0.3858 | No |
| 232 | FGF21 | na | 26242 | -0.118 | -0.3857 | No |
| 233 | PPARG | na | 26330 | -0.120 | -0.3857 | No |
| 234 | PDGFB | na | 26504 | -0.123 | -0.3881 | No |
| 235 | PIAS2 | na | 26547 | -0.124 | -0.3866 | No |
| 236 | STAT3 | na | 26744 | -0.129 | -0.3896 | No |
| 237 | ITGAV | na | 26862 | -0.132 | -0.3902 | No |
| 238 | WNT1 | na | 27000 | -0.135 | -0.3912 | No |
| 239 | FGF6 | na | 27010 | -0.135 | -0.3886 | No |
| 240 | PDGFRB | na | 27235 | -0.141 | -0.3921 | No |
| 241 | EP300 | na | 27396 | -0.145 | -0.3937 | No |
| 242 | TCF7L1 | na | 27666 | -0.152 | -0.3982 | Yes |
| 243 | MYC | na | 27707 | -0.153 | -0.3961 | Yes |
| 244 | FGF2 | na | 27758 | -0.154 | -0.3943 | Yes |
| 245 | FGF1 | na | 27813 | -0.156 | -0.3925 | Yes |
| 246 | SLC2A1 | na | 27886 | -0.157 | -0.3912 | Yes |
| 247 | STK36 | na | 27892 | -0.157 | -0.3880 | Yes |
| 248 | GLI2 | na | 27939 | -0.159 | -0.3859 | Yes |
| 249 | LAMC1 | na | 27992 | -0.160 | -0.3840 | Yes |
| 250 | LAMC2 | na | 28053 | -0.162 | -0.3823 | Yes |
| 251 | FAS | na | 28092 | -0.163 | -0.3799 | Yes |
| 252 | WNT10A | na | 28144 | -0.164 | -0.3779 | Yes |
| 253 | DVL2 | na | 28187 | -0.166 | -0.3755 | Yes |
| 254 | NFKB1 | na | 28241 | -0.168 | -0.3735 | Yes |
| 255 | BCL2L1 | na | 28417 | -0.173 | -0.3749 | Yes |
| 256 | ARAF | na | 28444 | -0.174 | -0.3719 | Yes |
| 257 | APC | na | 28648 | -0.180 | -0.3740 | Yes |
| 258 | MET | na | 28755 | -0.183 | -0.3731 | Yes |
| 259 | ARNT2 | na | 28765 | -0.184 | -0.3694 | Yes |
| 260 | CSF1R | na | 28825 | -0.186 | -0.3672 | Yes |
| 261 | MMP2 | na | 28890 | -0.188 | -0.3650 | Yes |
| 262 | WNT2B | na | 28930 | -0.189 | -0.3621 | Yes |
| 263 | CRKL | na | 29092 | -0.194 | -0.3626 | Yes |
| 264 | LAMA3 | na | 29367 | -0.202 | -0.3663 | Yes |
| 265 | WNT7B | na | 29404 | -0.203 | -0.3630 | Yes |
| 266 | ITGA6 | na | 29414 | -0.203 | -0.3589 | Yes |
| 267 | CDK2 | na | 29418 | -0.203 | -0.3546 | Yes |
| 268 | COL4A4 | na | 29690 | -0.213 | -0.3579 | Yes |
| 269 | ITGB1 | na | 29873 | -0.219 | -0.3585 | Yes |
| 270 | VEGFA | na | 29965 | -0.222 | -0.3564 | Yes |
| 271 | NRAS | na | 30019 | -0.224 | -0.3532 | Yes |
| 272 | FGF23 | na | 30092 | -0.227 | -0.3504 | Yes |
| 273 | TCF7 | na | 30107 | -0.227 | -0.3459 | Yes |
| 274 | MAP2K1 | na | 30150 | -0.229 | -0.3422 | Yes |
| 275 | LAMB3 | na | 30260 | -0.233 | -0.3404 | Yes |
| 276 | FZD10 | na | 30271 | -0.233 | -0.3357 | Yes |
| 277 | WNT8B | na | 30486 | -0.242 | -0.3368 | Yes |
| 278 | RAD51 | na | 30852 | -0.256 | -0.3419 | Yes |
| 279 | NFKB2 | na | 31039 | -0.266 | -0.3416 | Yes |
| 280 | RELA | na | 31135 | -0.271 | -0.3386 | Yes |
| 281 | PGF | na | 31338 | -0.282 | -0.3384 | Yes |
| 282 | NFKBIA | na | 31525 | -0.292 | -0.3376 | Yes |
| 283 | ZBTB16 | na | 31649 | -0.298 | -0.3348 | Yes |
| 284 | WNT9B | na | 31651 | -0.298 | -0.3284 | Yes |
| 285 | RUNX1 | na | 32001 | -0.317 | -0.3318 | Yes |
| 286 | WNT5B | na | 32169 | -0.328 | -0.3296 | Yes |
| 287 | WNT7A | na | 32275 | -0.335 | -0.3255 | Yes |
| 288 | RUNX1T1 | na | 32331 | -0.338 | -0.3198 | Yes |
| 289 | CSF2RA | na | 32334 | -0.339 | -0.3126 | Yes |
| 290 | FZD2 | na | 32361 | -0.340 | -0.3061 | Yes |
| 291 | TRAF5 | na | 32408 | -0.344 | -0.3001 | Yes |
| 292 | PTGS2 | na | 32511 | -0.351 | -0.2955 | Yes |
| 293 | AXIN2 | na | 32625 | -0.360 | -0.2911 | Yes |
| 294 | BCL2 | na | 32817 | -0.374 | -0.2887 | Yes |
| 295 | TGFB1 | na | 32868 | -0.377 | -0.2820 | Yes |
| 296 | GLI1 | na | 32884 | -0.379 | -0.2744 | Yes |
| 297 | TGFB3 | na | 33024 | -0.389 | -0.2701 | Yes |
| 298 | TRAF1 | na | 33097 | -0.397 | -0.2637 | Yes |
| 299 | WNT6 | na | 33111 | -0.398 | -0.2555 | Yes |
| 300 | COL4A6 | na | 33147 | -0.402 | -0.2479 | Yes |
| 301 | VEGFC | na | 33298 | -0.419 | -0.2433 | Yes |
| 302 | IL6 | na | 33353 | -0.425 | -0.2358 | Yes |
| 303 | LEF1 | na | 33408 | -0.432 | -0.2281 | Yes |
| 304 | BMP4 | na | 33633 | -0.460 | -0.2248 | Yes |
| 305 | CDKN1A | na | 33771 | -0.483 | -0.2184 | Yes |
| 306 | SHH | na | 33799 | -0.486 | -0.2088 | Yes |
| 307 | LAMA1 | na | 34028 | -0.528 | -0.2041 | Yes |
| 308 | NOS2 | na | 34080 | -0.537 | -0.1941 | Yes |
| 309 | EGLN3 | na | 34124 | -0.548 | -0.1836 | Yes |
| 310 | MMP9 | na | 34142 | -0.555 | -0.1722 | Yes |
| 311 | FZD7 | na | 34203 | -0.571 | -0.1617 | Yes |
| 312 | RXRG | na | 34332 | -0.610 | -0.1524 | Yes |
| 313 | FGF4 | na | 34351 | -0.616 | -0.1397 | Yes |
| 314 | MITF | na | 34465 | -0.663 | -0.1288 | Yes |
| 315 | WNT11 | na | 34512 | -0.683 | -0.1155 | Yes |
| 316 | NKX3-1 | na | 34541 | -0.702 | -0.1013 | Yes |
| 317 | MMP1 | na | 34577 | -0.723 | -0.0869 | Yes |
| 318 | ETS1 | na | 34588 | -0.730 | -0.0715 | Yes |
| 319 | BMP2 | na | 34656 | -0.831 | -0.0557 | Yes |
| 320 | CXCL8 | na | 34657 | -0.838 | -0.0377 | Yes |
| 321 | FGFR1 | na | 34693 | -0.924 | -0.0190 | Yes |
| 322 | AKT3 | na | 34696 | -0.932 | 0.0009 | Yes |
Table: GSEA details [plain text format]

  

Fig 2: KEGG\_PATHWAYS\_IN\_CANCER      
 Blue-Pink O' Gram in the Space of the Analyzed GeneSet

  

Fig 3: KEGG\_PATHWAYS\_IN\_CANCER: Random ES distribution      
 Gene set null distribution of ES for **KEGG\_PATHWAYS\_IN\_CANCER**
